# Machine-learning-guided optimization of cobalt doping in Sm(Fe,Ti,V)_12_-based magnets

**DOI:** 10.1080/14686996.2026.2691684

**Published:** 2026-07-07

**Authors:** Toni Subagja, Nikita Kulesh, Jiasheng Zhang, Andres Martin-Cid, Anton Bolyachkin, Xin Tang, Tadakatsu Ohkubo, Hossein Sepehri-Amin

**Affiliations:** a Research Center for Magnetic and Spintronic Materials, National Institute for Materials Science (NIMS), Tsukuba, Japan; b Graduate School of Science and Technology, University of Tsukuba, Tsukuba, Japan

**Keywords:** SmFe_12_-based magnets, coercivity, saturation magnetization, microstructure

## Abstract

Partial substitution of Fe with Co in Sm(Fe,Co)_12_-based compounds is known to enhance saturation magnetization but often causes significant coercivity degradation. To mitigate this coercivity loss, we compiled a dataset by mining published experimental data along with our in-house data and applied machine-learning techniques to search the optimal compositions. The machine learning predicts that coercivity degradation is suppressed when the Ti/V stabilizer ratio exceeds ~1.0. To validate experimentally, we prepared Sm_8_Zr_2_Fe_76.5-x_Co_x_Ti_8_V_5_Cu_0.5_(Ti-rich) and Sm_8_Zr_2_Fe_76.5-x_Co_x_Ti_5_V_8_Cu_0.5_(Ti-lean) rapidly solidified ribbons. Consistent with the machine-learning predictions, Ti-rich ribbons exhibited improved resistance to coercivity degradation compared with Ti-lean ribbons. Detailed microstructural characterization revealed that Co-doped ribbons maintained similar features, including comparable grain size and the presence of a Sm-rich intergranular phase regardless of different Ti/V ratios. However, the formation of secondary SmFe_2_ phases and the increase in twinned grains after Co doping were identified as factors contributing to coercivity reduction. Moreover, evaluation of intrinsic magnetic properties showed that the anisotropy field decreased more steeply in Ti-lean alloys than in Ti-rich alloys upon Co addition, consistent with the measured tendency of coercivity values. These results demonstrate that optimizing the matrix composition, specifically maintaining Ti-rich stabilizer, is a key strategy for advancing SmFe_12_-based permanent magnets, enabling a balance between high coercivity and magnetization for practical applications.

## IMPACT STATEMENT

Machine-learning-assisted alloy design in Co-doped Sm(Fe,Ti,V)_12_-based magnets revealed that maintaining a Ti/V stabilizer ratio above ~1 mitigates coercivity degradation while preserving high magnetization.

## GRAPHICAL ABSTRACT

**Figure:**
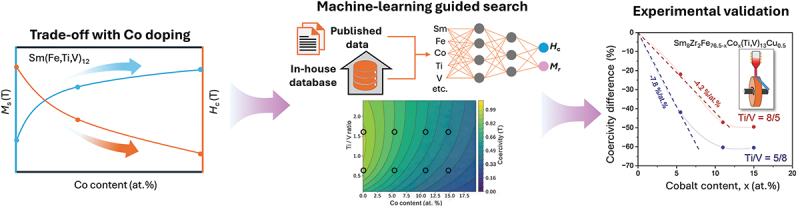

## 1. Introduction

Permanent magnets are used in energy-conversion applications such as wind-turbine generators, traction motors in hybrid/electric vehicles, and various household appliances [1,2]. Currently, (Nd,Dy)-Fe-B magnets remain the material of choice for these applications due to their large maximum energy product (*BH*)_max_ at operating temperatures of 393–473 K [3,4]. However, increasing demand and long-term sustainability concerns related to Dy and Nd are driving research to permanent magnets with reduced rare earth (RE) element content.

One candidate is the Fe-rich SmFe_12_-based compound with ThMn_12_-type crystal structure (1:12) [5]. This RE-lean compound (7.7 at.% RE) has attracted renewed research interest owing to its excellent intrinsic magnetic properties: a Curie temperature (*T*_c_) of 555 K, an anisotropy field (*μ*_0_*H*_a_) of 12 T, and a saturation magnetization (*μ*_0_*M*_s_) of 1.64 T at room temperature, as reported for SmFe_12_ thin film [6]. Despite these excellent properties, a key challenge is the thermodynamic instability of the SmFe_12_ phase in bulk form. To address this, elements such as Cr, V, Ti, Mo, W, Si, and Nb are needed to partially substitute for Fe in SmFe_12-x_M_x_ compounds [7–14]. However, introducing these stabilizing elements typically reduces magnetization [8,15].

Among the various stabilizing elements, Ti has received particular attention because it can effectively stabilize the ThMn_12_-type crystal structure at relatively low substitution levels. In Ti-only substituted SmFe_12-x_Ti_x_-type systems, the most effective Ti content reported for improving magnetic performance is on the order of 7.7 at.% [16,17], while still retaining a sufficiently large magnetization and anisotropy field [18,19]. Despite this advantage, achieving sufficiently high coercivity has shown limited success when Ti is the sole stabilizer in Sm(Fe,Ti)_12_-based magnets [10,13]. In contrast, V addition – either alone or in combination with Ti – has been reported to be important in enhancing coercivity [12,20]. Recent studies show that high coercivity is achieved only when a Sm-rich intergranular phase (IGP) forms and envelops the 1:12 grains, such a microstructure observed to date only in V-containing compounds [12,21–25]. The reduced Fe content in this intergranular region creates a weak or non-magnetic layer that effectively pins domain wall motion. Consequently, anisotropic Sm(Fe,V,Ti)_12_-based sintered magnets have exhibited coercivities in the range of 0.8–1.5 T [21–24]. However, their relatively low magnetization remains a major obstacle to practical application of these magnets. This leads to an optimization problem: identifying the optimal combination and minimal amount of stabilizing elements required to achieve high coercivity and high magnetization simultaneously.

Another common strategy to increase magnetization is to substitute Co for Fe in Sm(Fe,Co)_12_-based compounds [6,26–28]. Promising results have been demonstrated for thin films, Sm(Fe_0.8_Co_0.2_)_12_ exhibits an enhanced *μ*_0_*M*_s_ of 1.78 T, compared with 1.64 T for Co-free SmFe_12_, and its *T*_c_ raises to 859 K [6]. However, the drawback of this strategy is that Co adversely affects the coercivity. Zhang *et al*. reported that Co addition to Sm_8_(Fe_1-x_Co_x_)_73.5_Ti_8_V_8_Ga_0.5_Al_2_ reduced the coercivity from 1 T to 0.55 T when *x* increases from 0 to 0.05 [24]. Further studies showed that the effect of Co on intrinsic magnetic properties depends strongly on which stabilizing elements are used (e.g. Ti versus V) [26]. Therefore, although Co can improve magnetization, it makes the materials design more complicated. Exploring all possible combinations in Sm(Fe,Co,Ti,V,M)_12_ systems, alongside with processing conditions, becomes challenging using conventional experimental approaches alone.

To address this challenge, we employed machine learning (ML) to design the alloy and minimize coercivity loss caused by the Co addition. An artificial neural network was trained on a dataset compiled from both published literature and our in-house experimental results. The model predicts that the coercivity reduction associated with Co substitution in Sm(Fe,Ti,V)_12_-based compounds can be mitigated when the Ti/V ratio is higher. We validated this prediction experimentally using rapidly solidified melt-spun ribbons. To better understand why Co works more effectively at higher Ti/V ratios, we investigated both intrinsic magnetic properties and extrinsic contributions arising from microstructural features. Overall, this study demonstrates an ML-assisted alloy design can be an effective approach to improve the performance of SmFe_12_-based permanent magnets.

## 2. Experimental

**Figure 1.**
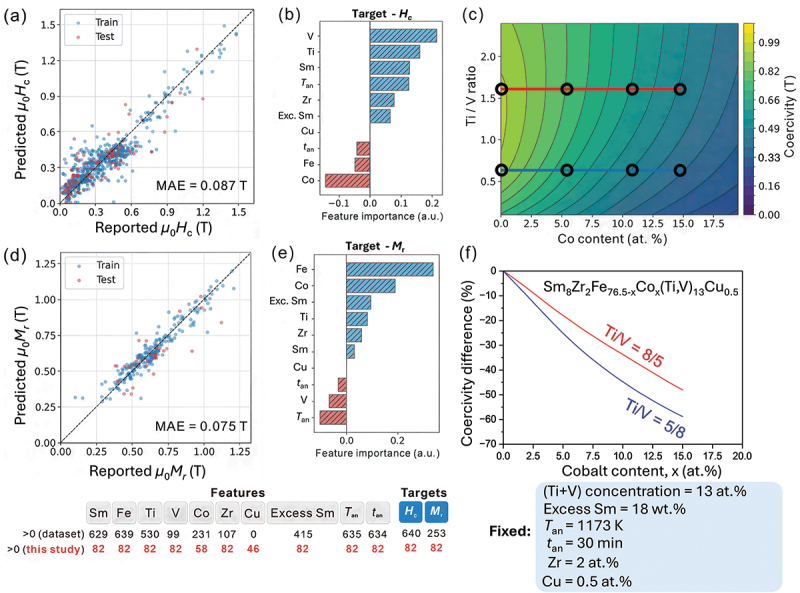
Performance and feature analysis of the MLP regressor trained to predict (a,b) coercivity and (d,e) remanence of SmFe_12_-based compounds. The bottom-left inset shows the number of nonzero inputs for features and targets in the original dataset from literature (black) and additional in-house dataset (red). (c) Predicted coercivity map as a function of Ti/V ratio and Co content, black circles indicate the compositions selected for experimental validation, and (f) calculated coercivity reduction profiles extracted from (c) for Ti/V = 8/5 and 5/8, illustrating the different sensitivities of coercivity reduction to Co addition at fixed total Ti+V concentration.

The coercivity of SmFe_1__2_-based melt-spun ribbons with varying Co contents and Ti/V ratios was analyzed using a combined dataset comprising in-house samples and those extracted from the literature [12]. A feedforward multi-layer perceptron (MLP) neural network with a single hidden layer (314 trainable parameters) was trained to predict coercivity and remanence based on compositional and processing parameters. The input features, and overall model performance are summarized in Fig. 1(a,d). Model performance was evaluated using leave-10-out cross-validation, and the reported mean absolute error (MAE) represents the average of fold-wise MAEs (60 folds). Feature importance was quantified as the sum of path weights in the trained MLP regressor, allowing identification of the dominant features affecting coercivity and remanence, as shown in Fig. 1(b,e). The trained model was applied to a grid of Co contents and Ti/V ratios of the alloy’s nominal compositions to obtain coercivity maps. An ensemble approach was employed to improve robustness of this mapping: 100 independent MLP regressors with identical architecture were trained on 85% bootstrap-resampled subsets of the dataset. Averaging the ensemble predictions from these regressors reduced fluctuations in the output, resulting in smoother and more reliable coercivity distribution map, while also enabling estimation of the prediction uncertainty [29].

Validations of ML predictions were conducted by synthesizing alloys with nominal compositions of Sm_8_Zr_2_Fe_76.5-x_Co_x_Ti_8_V_5_Cu_0.5_ and Sm_8_Zr_2_Fe_76.5-x_Co_x_Ti_5_V_8_Cu_0.5_ with *x* = 0, 5.5, 11, and 15 at.% using induction melting. It is important to note that, according to the dataset collected from literature, cobalt addition of around 15 at.% represents the practical upper limit for enhancing remanence while maintaining sufficiently high coercivity. For clarity, these compositions are hereafter referenced according to their respective Ti/V ratios. An 18 wt.% excess of Sm was added to compensate for its evaporation during melting. The intrinsic magnetic properties were evaluated using ingots of the corresponding compositions which were homogenized at 1100°C for 48 hours followed by pulverization to powders (<20 μm) and aligned under 3 T magnetic field and immobilized with an adhesive glue. Magnetization curves along and perpendicular to the axis of magnetic alignment were measured using a Physical Property Measurement System (PPMS; Quantum Design DynaCool) equipped with a vibrating sample magnetometer (VSM). The magnetic anisotropy field was estimated using the singular point detection (SPD) method from the second derivative of the initial magnetization curve measured along the hard axis of the aligned particles (Figure S6) [30,31]. *T*_c_ was determined from M – T curves measured under an external magnetic field of 0.05 T.

The extrinsic magnetic properties were evaluated using rapidly solidified ribbons produced by melt spinning onto a chrome-plated copper wheel rotating at 40 m/s speed under an Ar atmosphere. The as-quenched ribbons were annealed at 900°C for 30 minutes. Phase constitutions were examined by X-ray diffraction (XRD) using a Rigaku SmartLab diffractometer with Cr-Kα radiation (λ = 2.29 Å), and volume fractions were quantified through Rietveld refinement using the FullProf Suite [32]. Microstructural analysis was performed using a Carl Zeiss Cross-Beam 1540EsB scanning electron microscope (SEM). Scanning transmission electron microscopy (STEM) was carried out using a Titan G2 80–200 TEM equipped with a probe aberration corrector, while Energy-Dispersive X-ray Spectroscopy (EDS) data were collected using an FEI Super-X EDX detector. Transmission electron backscatter diffraction (t-EBSD) was conducted using Oxford EBSD detector. TEM specimens were prepared by lift-out method using a dual beam-focused ion beam (FEI Helios 5UX).

## 3. Results and discussion

The dataset of rapidly solidified SmFe_12_-based isotropic alloys acquired from the literature [12] served as a starting point for ML. However, this dataset lacked diversity in Ti/V ratios, samples containing Zr, and especially those with Cu. To improve the statistical representativeness, we iteratively expanded the dataset with 82 in-house melt-spun samples of (Sm,Zr)(Fe,Co,Ti,V,Cu)_12_ composition, Figure S1(a-b), targeted coercivity and remanence in an active-learning manner, meaning that the predictive ML model gets updated after new data points are evaluated experimentally and added to the dataset. The bottom-left inset of Fig. 1 summarizes the input features and target properties, showing the number of samples containing nonzero values in the original dataset from literature (black) and additional in-house dataset (red).

The MLP regressor was trained using the combined dataset, achieving mean absolute errors of 0.087 T for coercivity and 0.075 T for remanence (see training performance in Figure 1(a,d)). Feature importance analysis (Figure 1(b,e)) highlighted a clear trade-off: increasing the amount of V enhances coercivity but simultaneously reduces remanence, whereas higher Fe or Co content lead to the opposite effect. Notably, although Ti and Zr appear less critical for coercivity than V, they do not show detrimental effect on remanence. The MLP regressor was used to visualize these trends via coercivity maps and to identify compositional pathways for mitigating the negative impact of Co on coercivity.

The coercivity map was plotted under the following constraints. First, the total Ti + V concentration was fixed at 13 at.%, corresponding to the lowest Ti + V level in recent reports of high-coercivity SmFe_12_-based sintered magnets [21–25]. This choice was made because it provides a favorable balance between maintaining relatively high coercivity and still allowing an improvement in saturation magnetization. In addition, as shown in Supplementary, Figure S2(a,b), the difference between Ti-rich and Ti-lean becomes increasingly pronounced with Co addition, and this trend is clearly visible at 13 at.%, making this composition range particularly suitable for experimental validation. Moreover, considering the critical role of Cu in forming Sm-rich IGP in these magnets [21,22], a minor Cu content was fixed at 0.5 at.%. In light of the beneficial effect of Zr on stabilizing the 1:12 phase and enhancing saturation magnetization [28,33], Zr was set to 2 at.%. These constraints do not affect much the further analysis of the Ti/V ratio vs. Co trade-off (see coercivity maps in Supplementary, Figure S2(c-f), obtained with slight variations in Zr, Cu, Ti+V), and they may be relaxed in follow up studies to enable finer optimization as the dataset accumulates more samples. Finally, the following parameters were fixed for coercivity mapping: Sm content at 8 at.%, excess Sm at 18 wt.%, annealing temperature at 1173 K, and annealing time at 30 minutes.

Figure 1(c) shows a coercivity map of the Sm_8_Zr_2_Fe_76.5-x_Co_x_(Ti,V)_13_Cu_0.5_ compound as a function of the Ti/V ratio and Co content under the constraints described above. The map reveals that higher coercivity can be achieved upon Co addition for compositions consisting of larger Ti/V ratio. The observed flattening and downward shift of the coercivity contours with increasing Co content further illustrate the growing importance of maintaining higher Ti/V ratios to preserve coercivity in Co-rich alloys. Figure 1(f) quantitatively illustrates the coercivity loss induced by Co addition at two different Ti/V ratios, 5/8 and 8/5. These ratios were chosen for synthesizing a series of Sm_8_Zr_2_Fe_76.5-x_Co_x_(Ti,V)_13_Cu_0.5_ samples with *×*=0, 5.5, 11, and 15 at.% (indicated by black circles in Figure 1(c)), enabling a systematic investigation of the physical origins of the observed trend.

**Figure 2.**
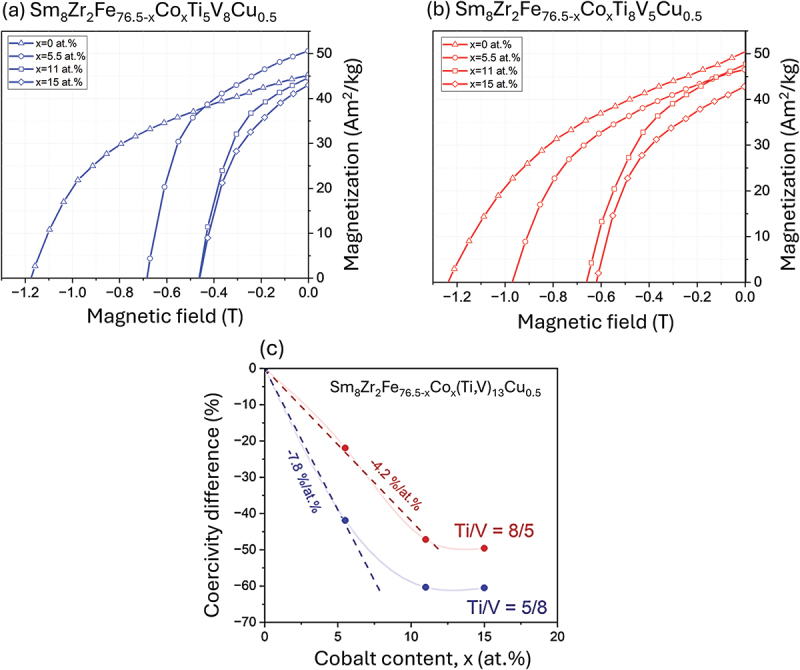
Room-temperature demagnetization curves of Sm_8_Zr_2_Fe_76.5-x_Co_x_(Ti,V)_13_Cu_0.5_ ribbons with different Ti/V ratios of (a) 5/8 and (b) 8/5 for varying Co contents. (c) Corresponding coercivity difference as a function of Co content.

Figure 2 shows the room-temperature demagnetization curves for two series of Sm_8_Zr_2_Fe_76.5-x_Co_x_(Ti,V)_13_Cu_0.5_ ribbons with *×*=0, 5.5, 11, and 15 at.%, corresponding to (a) Ti/V = 5/8 and (b) Ti/V = 8/5. In both series, coercivity decreases with increasing Co content, as summarized in Figure 2(c). The Ti-lean samples (Ti/V = 5/8) exhibit a rapid reduction in coercivity – about 7.8% per 1 at.% Co at low concentrations – reaching nearly 60% coercivity loss for Co > 10 at.%. In contrast, the Ti-rich samples (Ti/V = 8/5) demonstrate a better resistance to Co-induced coercivity deterioration: 4.2% coercivity loss per 1 at.% Co at low concentrations with a higher asymptotic retention of about 50%. These results reasonably agree with ML predictions in Fig. 1(f) where the initial slopes of coercivity decrease are 3.5% and 5.0% per 1 at.% Co for Ti/V = 8/5 and Ti/V = 5/8, respectively. Thus, a higher Ti/V ratio is confirmed to be preferable for maintaining coercivity in SmFe_12_-based allows upon Co addition, particularly at Co concentrations below 10 at.%. The next question is which extrinsic (microstructural) and intrinsic factors are responsible for this observed coercivity behavior.

**Figure 3.**
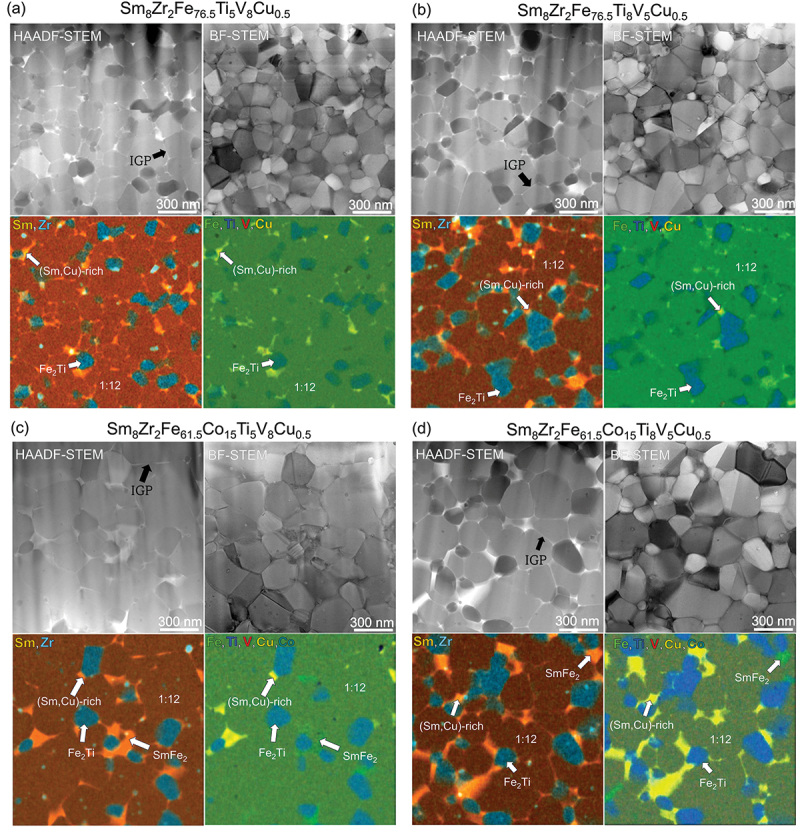
HAADF-STEM, BF-STEM, and STEM-EDS elemental maps for Co-free Sm_8_Zr_2_Fe_76.5_(Ti,V)_13_Cu_0.5_ ribbons with (a) Ti/V = 5/8 and (b) Ti/V = 8/5 along with Co-containing Sm_8_Zr_2_Fe_61.5_Co_15_(Ti,V)_13_Cu_0.5_ ribbons with (c) Ti/V = 5/8 and (d) Ti/V = 8/5.

Figure 3 presents the general microstructures of Ti-lean (Ti/V = 5/8) and Ti-rich (Ti/V = 8/5) Sm_8_Zr_2_Fe_76.5-x_Co_x_(Ti,V)_13_Cu_0.5_ ribbons, shown both without Co (a,b) and with 15 at.% Co (c,d), including high angle annular dark field (HAADF)-STEM images, bright-field (BF)-STEM images, and corresponding STEM-EDS elemental maps. The mean grain sizes of the Co-free samples were found to be comparable − 150 (50) nm for Ti/V = 5/8 and 180 (70) nm for Ti/V = 8/5. Standard deviations are given in parentheses, while the entire grain size histograms are available in Figure S3.

Upon Co addition, the grain size increases to 240 (100) nm and 230 (90) nm for Ti/V = 5/8 and 8/5, respectively, again without a significant difference between the two Ti/V ratios. Thus, while the grain size increase induced by Co addition does play a role in lowering coercivity [34–36], it can hardly explain the variations in coercivity observed between the samples with different Ti/V ratios.

Another extrinsic factor that can influence coercivity is the presence and morphology of secondary phases. STEM-EDS maps of the constituent elements (Figure 3) indicate that all ribbons contain the (Fe,Co)_2_(Ti,Zr) phase as well as thick (Sm,Cu)-rich intergranular phase. The amount of (Fe,Co)_2_(Ti,Zr) is apparently higher in the Ti-rich samples. However, these two secondary phases seem to be nonmagnetic at room temperature according to their chemical compositions [37–39]. As for magnetic secondary phases, *α*-Fe was not detected in all samples, as additionally confirmed by the XRD results (Fig. S4). In contrast, Sm(Fe,Co)_2_ magnetic phase appeared upon Co addition (see Figure 3(c,d)), that should contribute in the observed coercivity deterioration. However, according to the Rietveld refinement analysis resulting in table S1, its amount seems to be comparable in samples with different Ti/V ratios. It is also worth noting that some 1:12 grains are well isolated from each other in HAADF-STEM images (Figure 3) that manifests the formation of a thin Sm-rich intergranular phase, which could be magnetic depending on its chemical composition.

**Figure 4.**
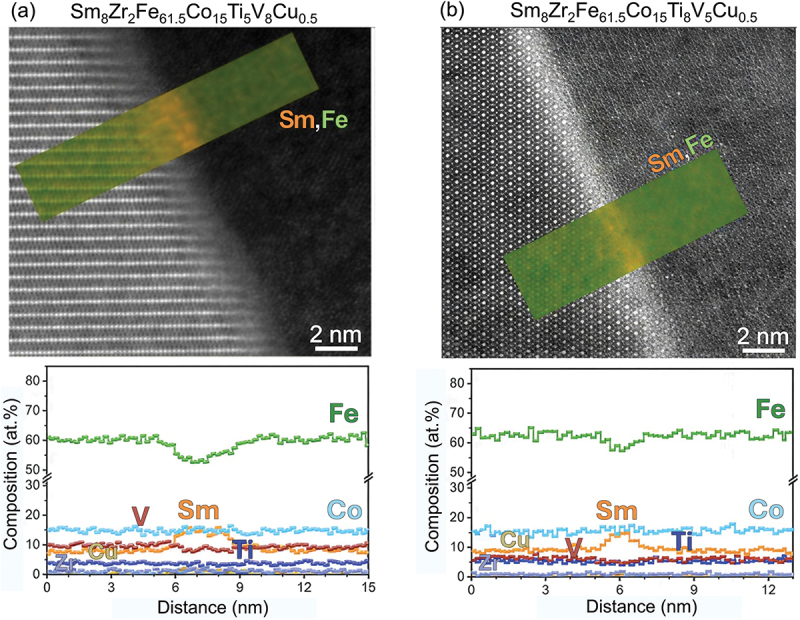
High magnification HAADF-STEM images, superimposed STEM-EDS maps of Sm and Fe, and corresponding composition line profiles of constituent elements obtained from thin IGP of (a) Sm_8_Zr_2_Fe_61.5_Co_15_Ti_5_V_8_Cu_0.5_ and (b) Sm_8_Zr_2_Fe_61.5_Co_15_Ti_8_V_5_Cu_0.5_ ribbons.

This thin IGP was examined for Sm_8_Zr_2_Fe_61.5_Co_15_Ti_5_V_8_Cu_0.5_ ribbons at higher magnification incorporating STEM-EDS, with representative results shown in Fig. 4 for (a) Ti/V = 5/8 and (b) Ti/V = 8/5. In both samples, the amorphous Sm-rich IGP with a thickness of ~3 nm is observed. In the Ti-lean sample (Ti/V = 5/8), the IGP contains 15.1 at.% Sm and 68.4 at.% Fe+Co, while in the Ti-rich sample (Ti/V = 8/5) the corresponding values are 14.4 at.% and 74.0 at.%, respectively. Such a high Fe+Co content suggests that the IGP is likely magnetic, and thus the 1:12 grains are expected to be exchange‑coupled. Nevertheless, the IGP compositions in the two samples are quite similar, with the Ti‑rich sample even showing a slightly higher Fe+Co content – opposite to its higher coercivity. Therefore, the chemistry of thin IGP can also be ruled out as a potential reason behind the effect of Ti/V ratio on the coercivity (Figure 2(c)). The last extrinsic factor to consider, which is specific for 1:12 magnets, is the presence of crystallographic twins. These are clearly visible in BF‑STEM images for all samples (Figure 3) as regions with distinct contrast in some grains.

**Figure 5.**
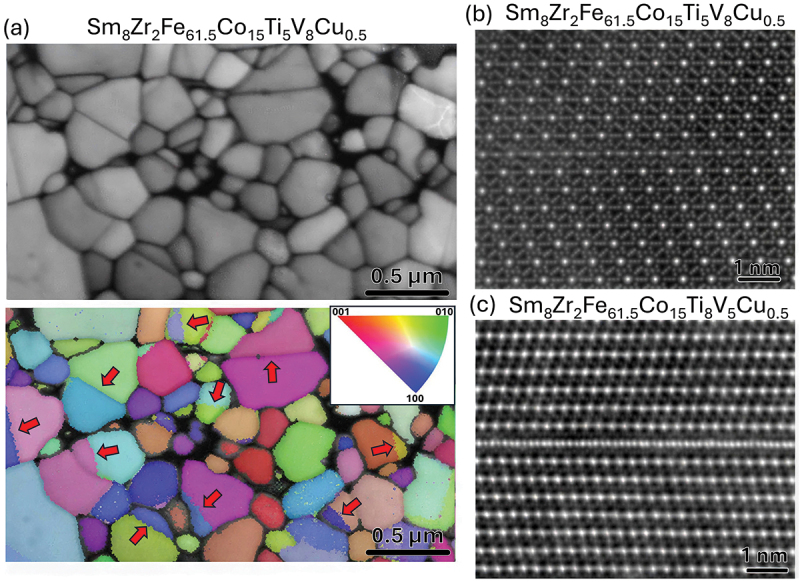
(a) Transmission EBSD of the Sm_8_Zr_2_Fe_61.5_Co_15_Ti_5_V_8_Cu_0.5_ ribbon showing the band-contrast image (top) and inverse Pole figure (bottom) of 1:12 grains, with crystallographic twins highlighted by red arrows. High magnification HAADF-STEM images of representative twin boundary regions in (b) Sm_8_Zr_2_Fe_61.5_Co_15_Ti_5_V_8_Cu_0.5_ and (c) Sm_8_Zr_2_Fe_61.5_Co_15_Ti_8_V_5_Cu_0.5_ samples, indicating the distinct atomic projections across the twin boundary.

Crystallographic twins were directly confirmed by the transmission EBSD, as demonstrated for the Sm_8_Zr_2_Fe_61.5_Co_15_Ti_5_V_8_Cu_0.5_ ribbon in Figure 5(a). Twins were observed in all samples, and STEM analysis indicates that the fraction of twinned grains is comparable between the Ti-lean and Ti-rich compositions. For the Co-free ribbons, the twin fractions were approximately 23% and 26% for the Ti-lean and Ti-rich samples, respectively. Upon 15 at.% Co addition, these values increased to about 33% and 31%, respectively, indicating no clear correlation between twin fraction and Ti/V ratio. Interestingly, such extensive twinning occurs despite the relatively fine mean grain size (150–240 nm), raising the question of whether grain refinement is truly effective in suppressing twin formation and what the actual grain-size threshold for this mechanism might be [26].

Beyond the overall density of twins, the atomic configuration at the twin boundary can also influence the local nucleation and/or pinning of magnetic domain walls and thus affect coercivity. C.E. Patrick *et al*. reported that the magnetocrystalline anisotropy at a twin boundary can vary significantly depending on its atomic arrangement [40]. High-resolution HAADF-STEM images in Figure 5(b,c) indeed reveal various scenarios of atomic arrangement across twin boundaries in samples with different Ti/V ratios. However, available statistics are insufficient to establish any clear correlation of this twin boundary feature with composition, and with Ti/V ratio. Although the increase in the areal fraction of twinned grains and the formation of Sm(Fe,Co)_2_ phases induced by Co addition can reduce coercivity, an important question remains: how do the intrinsic magnetic properties of the main 1:12 phase evolve with Co addition in samples with different Ti/V ratios?

**Figure 6.**
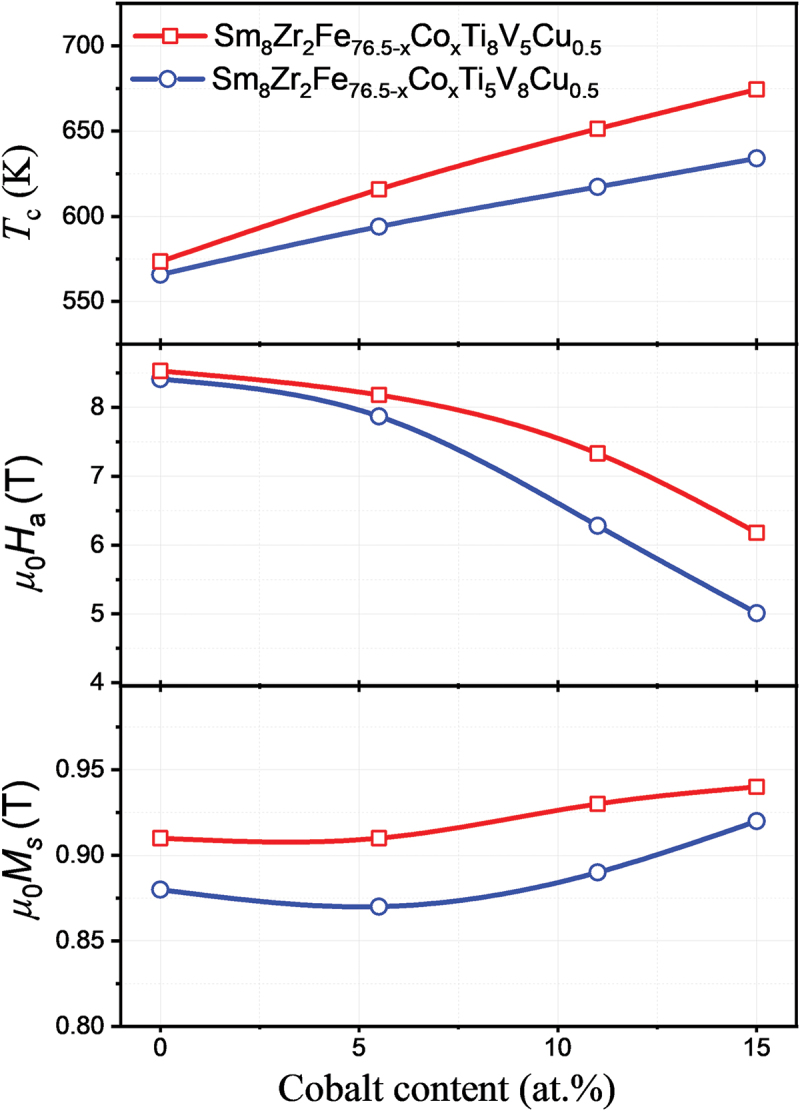
Curie temperature (*T*_c_), anisotropy field (*μ*_0_*H*_a_) and saturation magnetization (*μ*_0_*M*_s_) as functions of Co content for ingot samples of Sm_8_Zr_2_Fe_76.5-x_Co_x_Ti_8_V_5_Cu_0.5_ and Sm_8_Zr_2_Fe_76.5-x_Co_x_Ti_5_V_8_Cu_0.5_.

The intrinsic magnetic properties – Curie temperature (*T*_c_), magnetic anisotropy field (*H*_a_), and saturation magnetization (*M*_s_) – were evaluated for ingots of Sm_8_Zr_2_Fe_76.5-x_Co_x_(Ti,V)_13_Cu_0.5_ alloys with *x* = 0, 5.5, 11, and 15 at.%. All measurements were conducted on magnetically aligned powders that were pulverised from ingots that had been optimally annealed. Although melt-spun ribbons undergo an additional processing step compared to ingots, evaluating intrinsic magnetic properties, particularly the anisotropy field, on magnetically aligned single-crystalline powders provide greater accuracy in assessing how these properties evolve with Co addition across different Ti/V ratios. It is also important to note that the Ti/V ratio remains similar in the matrix phase of both melt-spun ribbons and ingots, as shown in Table 1 and Figure 4. The results are summarized in Figure 6, with processing details provided in Figs. S5 and S6. Matrix compositions for the *×*=0 and 15 at.% Co ingots were obtained by EDS and are listed in Table 1 along with the corresponding intrinsic magnetic properties. The actual Ti/V ratios in Ti-rich and Ti-lean samples are approximately 1.0 and 0.5, respectively, that deviate from the nominal ratios of 1.6 and 0.625. Importantly, Co addition does not alter the Ti/V ratio, indicating the observed trends in intrinsic properties are primarily determined by Co substitution for Fe under different stabilizer (Ti/V) ratios.

**Table 1.** Matrix compositions and intrinsic magnetic properties of Ti-rich and Ti-lean ingot samples of Sm_8_Zr_2_Fe_76.5-x_Co_x_(Ti,V)_13_Cu_0.5_ without Co and with 15 at.% Co. For each composition, the Curie temperature (*T*_c_), anisotropy field (*μ*_0_*H*_a_), and saturation magnetization (*μ*_0_*M*_s_) are listed.

Samples		Matrix composition	*T*_c_ (K)	*μ*_0_*H*_a_ (T)	*μ*_0_*M*_s_ (T)
Ti-rich	Co-free	Sm_8.74_Zr_1.02_Fe_75.05_Ti_7.38_V_7.81_	573	8.3	0.91
	15 at.% Co	Sm_8.61_Zr_0.98_Fe_61.81_ Co_12.87_Ti_8.04_V_7.69_	675	6.1	0.94
Ti-lean	Co-free	Sm_8.78_Zr_0.94_Fe_72.93_Ti_5.37_V_11.98_	565	7.9	0.88
	15 at.% Co	Sm_8.16_Zr_1.36_Fe_60.25_ Co_12.67_Ti_5.87_V_11.69_	634	4.6	0.92

As the Co content increases, *T*_c_ rises steadily for both Ti-rich and Ti-lean compositions, the Curie temperature was estimated from the inflection point of the *M-T* curve using the second-derivative method [41]. However, the rate of this increase differs: Ti-rich alloys exhibit a more significant enhancement, with *T*_c_ increasing from 573 K (x = 0) to 675 K (x = 15 at.% Co), whereas Ti-lean alloys show a more moderate rise from 566 K to 634 K across the same Co range. The more pronounced *T*_c_ response in Ti-rich compositions suggests that the role of Co is more effective in Ti-stabilized compounds, consistent with reports that Co exhibits a stronger effect with Ti than with V in ThMn_12_-type systems [18,26].

The saturation magnetization did not vary as remarkably as *T*_c_. In the Ti-rich alloys, *μ*_0_*M*_s_ increases slightly from 0.91 to 0.94 T as Co content reaches 15 at.%, whereas in the Ti-lean alloys it rises from 0.88 to 0.92 T. These values were corrected for the volume fractions of secondary phases, which were quantified by Rietveld refinement of XRD results (Figs. S7 and S8). The refinement confirmed that the 1:12 phase was dominant in all ingots, while Fe_2_Ti, α-Fe, SmCu, and SmFe_2_ presented only in minor or trace amounts.

In contrast to the beneficial role of Co element in *T*_c_ and *M*_s_, its addition leads to a steady decrease in the anisotropy field in Sm_8_Zr_2_Fe_76.5-x_Co_x_(Ti,V)_13_Cu_0.5_ alloy series. Notably, the magnitude of this reduction is composition-dependent. In the Ti-rich samples, *μ*_0_*H*_a_ decreases from 8.3 T (x = 0) to 6.2 T (x = 15 at.%), suggesting that moderate Co substitution can still preserve a relatively high anisotropy field. In contrast, the Ti-lean samples exhibit a more pronounced decrease in *μ*_0_*H*_a_ (from 7.7 T to 4.6 T). This behaviour is consistent with previous work showing that the Co-induced anisotropy reduction is more severe when V is the sole stabilizing element than in Ti-stabilized ThMn_12_-type compounds [26].

The variation of *H*_a_ upon Co addition qualitatively reproduces the coercivity trends observed in the studied samples across different Ti/V ratios (Figure 2(c)). However, this does not imply that microstructural factors are negligible. Factors such as the increased twin density and the formation of the Sm(Fe,Co)_2_ phase in Co-doped samples, compared with the Co-free samples, may also influence the coercivity. Therefore, the sensitivity of *H*_c_ to the Ti/V ratio primarily originates from changes in the magnetocrystalline anisotropy of the 1:12 main phase, while microstructural features, including twinned grains and secondary phases, are also likely to contribute. This indicates that the ML regressor successfully learned from rather scattered experimental data how the intrinsic magnetic properties of 1:12 compounds vary with composition. To further improve prediction performance, the experimental dataset can be complemented with microstructural information as well as theoretical data on intrinsic and thermodynamic properties of SmFe_12_-based compounds – such as results from high-throughput first-principles simulations [42,43] – in a manner similar to recent work on Nd-Fe-B magnets [44]. Such data assimilation will enable less constrained and more efficient compositional optimization of both magnetization and coercivity in prospective 1:12 magnets.

## 4. Conclusion

This study systematically investigates how cobalt substitution affects the coercivity of Sm(Fe,Ti,V)_12_-based compounds, focusing particularly on its interplay with different Ti/V ratios. Our machine learning and experiments collectively concludes that maintaining a Ti/V ≳ 1 is an effective pathway to achieve practically high coercivity with improved magnetization when Co is alloyed in SmFe_12_-based systems.

Comprehensive microstructural characterization indicates that grain sizes and intergranular-phase chemistry remained comparable between the Ti-rich and Ti-lean Co-doped samples. However, the formation of secondary Sm(Fe,Co)_2_ phases and the increased fraction of twinned grains after Co addition were identified as microstructural factors contributing to coercivity degradation. In addition to these microstructural effects, the coercivity behavior was strongly influenced by changes in the magnetocrystalline anisotropy of the 1:12 matrix for different Ti/V ratios. Specifically, Co-induced reduction of the anisotropy field was more pronounced in Ti-lean compositions than in Ti-rich compositions, explaining the superior coercivity retention of Ti-rich alloys. Furthermore, Co is more effective in increasing *T*_c_ and magnetization, while causing a smaller reduction in anisotropy field, in the Ti-rich composition. This behaviour is promising for achieving an improved balance between high coercivity and high remanent magnetization in bulk magnets, thereby facilitating the development of advanced permanent magnets for demanding applications such as electric vehicles and wind-energy systems.

## Supplementary Material

Supplemental Material

## References

[1] Coey JMD. Perspective and prospects for rare earth permanent magnets. Eng. 2020;6(2):119–10. doi: 10.1016/j.eng.2018.11.034

[2] Lixandru A, Venkatesan P, Jönsson C, et al. Identification and recovery of rare-earth permanent magnets from waste electrical and electronic equipment. Waste Manag. 2017;68:482–489. doi: 10.1016/j.wasman.2017.07.028 28751173

[3] Sagawa M, Fujimura S, Togawa N, et al. New material for permanent magnets on a base of Nd and Fe (invited). J Appl Phys. 1984;55(6):2083–2087. doi: 10.1063/1.333572

[4] Sagawa M, Fujimura S, Yamamoto H, et al. Permanent magnet materials based on the rare earth-iron-boron tetragonal compounds (invited). IEEE Trans Magn. 1984;20(5):1584–1589. doi: 10.1109/TMAG.1984.1063214

[5] Ohashi K, Tawara Y, Osugi R, et al. Magnetic properties of Fe-rich rare-earth intermetallic compounds with a ThMn12 structure. J Appl Phys. 1988;64(10):5714–5716. doi: 10.1063/1.342235

[6] Hirayama Y, Takahashi YK, Hirosawa S, et al. Intrinsic hard magnetic properties of Sm(Fe1−xCox)12 compound with the ThMn12 structure. Scr Mater. 2017;138:62–65. doi: 10.1016/j.scriptamat.2017.05.029

[7] Hu B-P, Hong-Shuo Li JPG, Gavigan JP, et al. Intrinsic magnetic properties of the iron-rich ThMn12-structure alloys R(Fe11Ti); R=Y, Nd, Sm, Gd, Tb, Dy, Ho, Er, Tm and Lu. J Phys Condens Matter. 1989;1(4):755. doi: 10.1088/0953-8984/1/4/009

[8] Coehoorn R. Electronic structure and magnetism of transition-metal-stabilized YFe12xMx intermetallic compounds. Phys Rev B. 1990;41(17):11790–11797. doi: 10.1103/PhysRevB.41.11790 9993626

[9] Li HS, Coey JMD. Chapter 1 magnetic properties of ternary rare-earth transition-metal compounds. In: K.H.J. Buschow, editor. Handbook of magnetic materials. Vol. 6. Elsevier; 1991. p. 1–83. doi: 10.1016/S1567-2719(05)80055-1

[10] Buschow KHJ. Permanent magnet materials based on tetragonal rare earth compounds of the type RFe12−xMx. J Magn Magn Mater. 1991;100(1):79–89. doi: 10.1016/0304-8853(91)90813-P

[11] De Mooij DB, Buschow KHJ. Some novel ternary ThMn12-type compounds. J Common Met. 1988;136(2):207–215. doi: 10.1016/0022-5088(88)90424-9

[12] Tang X, Li J, Srinithi AK, et al. Role of V on the coercivity of SmFe12-based melt-spun ribbons revealed by machine learning and microstructure characterizations. Scr Mater. 2021;200:113925. doi: 10.1016/j.scriptamat.2021.113925

[13] Okada M, Kojima A, Yamagishi K, et al. High coercivity in melt-spun SmFe10(Ti,M)2 ribbons (M=V/Cr/Mn/Mo). IEEE Trans Magn. 1990;26(5):1376–1378. doi: 10.1109/20.104383

[14] Dirba I, Harashima Y, Sepehri-Amin H, et al. Thermal decomposition of ThMn12-type phase and its optimum stabilizing elements in SmFe12-based alloys. J Alloys Compd. 2020;813:152224. doi: 10.1016/j.jallcom.2019.152224

[15] Verhoef R, de Boer FR, Zhi-Dong Z, et al. Cr V, Mo. Moment reduction in RFe12-xTx compounds (R=Gd, Y and T=Ti. W). J Magn Magn Mater. 1988;75(3):319–322. doi: 10.1016/0304-8853(88)90037-6

[16] Nan-Xian C, Shi-Qiang H, Yu W, et al. Phase stability and site preference of Sm(Fe,T)12. J Magn Magn Mater. 2001;233(3):169–180. doi: 10.1016/S0304-8853(01)00251-7

[17] Saito T, Watanabe F, Nishio-Hamane D. Magnetic properties of SmFe12-based magnets produced by spark plasma sintering method. J Alloys Compd. 2019;773:1018–1022. doi: 10.1016/j.jallcom.2018.09.297

[18] Kuno T, Suzuki S, Urushibata K, et al. (Sm,Zr)(Fe,Co)11.0–11.5Ti1.0–0.5 compounds as new permanent magnet materials. AIP Adv. 2016;6(2):025221. doi: 10.1063/1.4943051

[19] Kim HT, Kim YB, Kim CS, et al. Magnetocrystalline anisotropy of (Sm0.5RE0.5)Fe11Ti compounds (Re=Ce, Pr, Nd, Sm, Gd and Tb). J Magn Magn Mater. 1996;152(3):387–390. doi: 10.1016/0304-8853(95)00464-5

[20] Pinkerton FE, Van Wingerden DJ. Magnetic hardening of SmFe10V2 by melt-spinning. IEEE Trans Magn. 1989;25(5):3306–3308. doi: 10.1109/20.42285

[21] Srinithi AK, Tang X, Sepehri-Amin H, et al. High-coercivity SmFe12-based anisotropic sintered magnets by Cu addition. Acta Mater. 2023;256:119111. doi: 10.1016/j.actamat.2023.119111

[22] Zhang JS, Tang X, Ohkubo T, et al. Mechanism of continuous intergranular phase formation in Sm(Fe,Ti,V)12-based sintered magnets by post-sinter annealing. Acta Mater. 2025;294:121160. doi: 10.1016/j.actamat.2025.121160

[23] Zhang JS, Tang X, Sepehri-Amin H, et al. Origin of coercivity in an anisotropic Sm(Fe,Ti,V)12-based sintered magnet. Acta Mater. 2021;217:117161. doi: 10.1016/j.actamat.2021.117161

[24] Zhang JS, Tang X, Bolyachkin A, et al. Microstructure and extrinsic magnetic properties of anisotropic Sm(Fe,Ti,V)12-based sintered magnets. Acta Mater. 2022;238:118228. doi: 10.1016/j.actamat.2022.118228

[25] Otsuka K, Kamata M, Nomura T, et al. Coercivities of Sm–Fe–M sintered magnets with ThMn12-type structure (M = Ti, V). Mater Trans. 2021;62(6):887–891. doi: 10.2320/matertrans.MT-MBW2020003

[26] Tozman P, Hrkac G, Patrick CE, et al. Effect of Co on twin formation and magnetic properties of Sm(Fe,Ti,V)12 alloys. Scr Mater. 2025;258:116491. doi: 10.1016/j.scriptamat.2024.116491

[27] Tozman P, Sepehri-Amin H, Takahashi YK, et al. Intrinsic magnetic properties of Sm(Fe1-xCox)11Ti and Zr-substituted Sm1-yZry(Fe0.8Co0.2)11.5Ti0.5 compounds with ThMn12 structure toward the development of permanent magnets. Acta Mater. 2018;153:354–363. doi: 10.1016/j.actamat.2018.05.008

[28] Tozman P, Takahashi YK, Sepehri-Amin H, et al. The effect of Zr substitution on saturation magnetization in (Sm1-xZrx)(Fe0.8Co0.2)12 compound with the ThMn12 structure. Acta Mater. 2019;178:114–121. doi: 10.1016/j.actamat.2019.08.003

[29] Lambert B, Forbes F, Doyle S, et al. Trustworthy clinical AI solutions: a unified review of uncertainty quantification in deep learning models for medical image analysis. Artif Intell Med. 2024;150:102830. doi: 10.1016/j.artmed.2024.102830 38553168

[30] Aubert A, Skokov K, Gutfleisch O. Determining the anisotropy field of permanent magnets: a comparison of current methodologies. J Magn Magn Mater. 2025;634:173566. doi: 10.1016/j.jmmm.2025.173566

[31] Cabassi R. Singular point detection for characterization of polycrystalline permanent magnets. Measurement. 2020;160:107830. doi: 10.1016/j.measurement.2020.107830

[32] Rodríguez-Carvajal J. Recent advances in magnetic structure determination by neutron powder diffraction. Phys B: Condens Matter. 1993;192(1):55–69. doi: 10.1016/0921-4526(93)90108-I

[33] Landa A, Söderlind P, Moore EE, et al. Thermodynamics and magnetism of SmFe12 compound doped with Zr, Ce, Co and Ni: an ab initio study. Metals. 2024;14(1):59. doi: 10.3390/met14010059

[34] Uestuener K, Katter M, Rodewald W. Dependence of the mean grain size and coercivity of sintered Nd–Fe–B magnets on the initial powder particle size. IEEE Trans Magn. 2006;42(10):2897–2899. doi: 10.1109/TMAG.2006.879889

[35] Ramesh R, Srikrishna K. Magnetization reversal in nucleation controlled magnets. I Theory J Appl Phys. 1988;64(11):6406–6415. doi: 10.1063/1.342054

[36] Li WF, Ohkubo T, Hono K, et al. The origin of coercivity decrease in fine grained Nd–Fe–B sintered magnets. Curr Perspect Spintron. 2009;321(8):1100–1105. doi: 10.1016/j.jmmm.2008.10.032

[37] Pelloth J, Brand RA, Keune W. Local magnetic properties of the Fe2Ti Laves phase. Int Conf Magn. 1995;140–144:59–60. doi: 10.1016/0304-8853(94)00836-1

[38] Wertheim GK, Buchanan DNE, Wernick JH. Magnetic properties of inequivalent iron atoms in Fe2Ti. Solid State Commun. 1970;8(24):2173–2176. doi: 10.1016/0038-1098(70)90244-9

[39] Hoffmann E, Entel P, Wassermann E, et al. Electronic structure and magnetovolume instabilities of the hexagonal laves phase compound Fe2Ti. J Phys IV Proc. 1995;5(C2):C2–117. doi: 10.1051/jp4:1995218

[40] Patrick CE. Anisotropy at twin interfaces in RT12 (R=rare earth; T=transition metal) magnets. Phys Rev B. 2025;112(5):054446. doi: 10.1103/syc2-qzdf

[41] Fabian K, Shcherbakov VP, McEnroe SA. Measuring the Curie temperature. Geochem Geophys Geosyst. 2013;14(4):947–961. doi: 10.1029/2012GC004440

[42] Batnyam N, Odkhuu D. First-principles study of structural stability and intrinsic magnetic properties of SmFe11Ti-type alloys with Ni addition. AIP Adv. 2025;15(3):035114. doi: 10.1063/9.0000860

[43] Nguyen DN, Dam HC. Machine learning-aided genetic algorithm in investigating the structure–property relationship of SmFe12-based structures. J Appl Phys. 2023;133(6):063902. doi: 10.1063/5.0134821

[44] Kovacs A, Fischbacher J, Oezelt H, et al. Physics-informed machine learning combining experiment and simulation for the design of neodymium-iron-boron permanent magnets with reduced critical-elements content. Front Mater. 2023;9:9. doi: 10.3389/fmats.2022.1094055

