# Supplementary material for: Machine-learning-guided optimization of cobalt doping in Sm(Fe,Ti,V)12-based magnets
Source: Sci Technol Adv Mater. 2026 Jul 7;27(1):2691684. doi: 10.1080/14686996.2026.2691684 (PMC13347853; doi:10.1080/14686996.2026.2691684)
Supplement: Supplemental Material [file TSTA_A_2691684_SM0577.docx]

**Supplementary Information**

**Machine-learning-guided optimization of cobalt doping in Sm(Fe,Ti,V)_12_-based magnets**

Toni Subagja^a,b^, Nikita Kulesh^a^, Jiasheng Zhang^a^, Andres Martin-Cid^a^, Anton Bolyachkin^a^, Xin Tang^a^, Tadakatsu Ohkubo^a^, Hossein Sepehri-Amin^a,b,*^

^a^*Research Center for Magnetic and Spintronic materials,* *National Institute for Materials Science (NIMS), Tsukuba, Japan;* ^b^*Graduate School of Science and Technology, University of Tsukuba, Tsukuba, Japan*

^*^Corresponding author: [h.sepehriamin@nims.go.jp](mailto:h.sepehriamin@nims.go.jp)

*
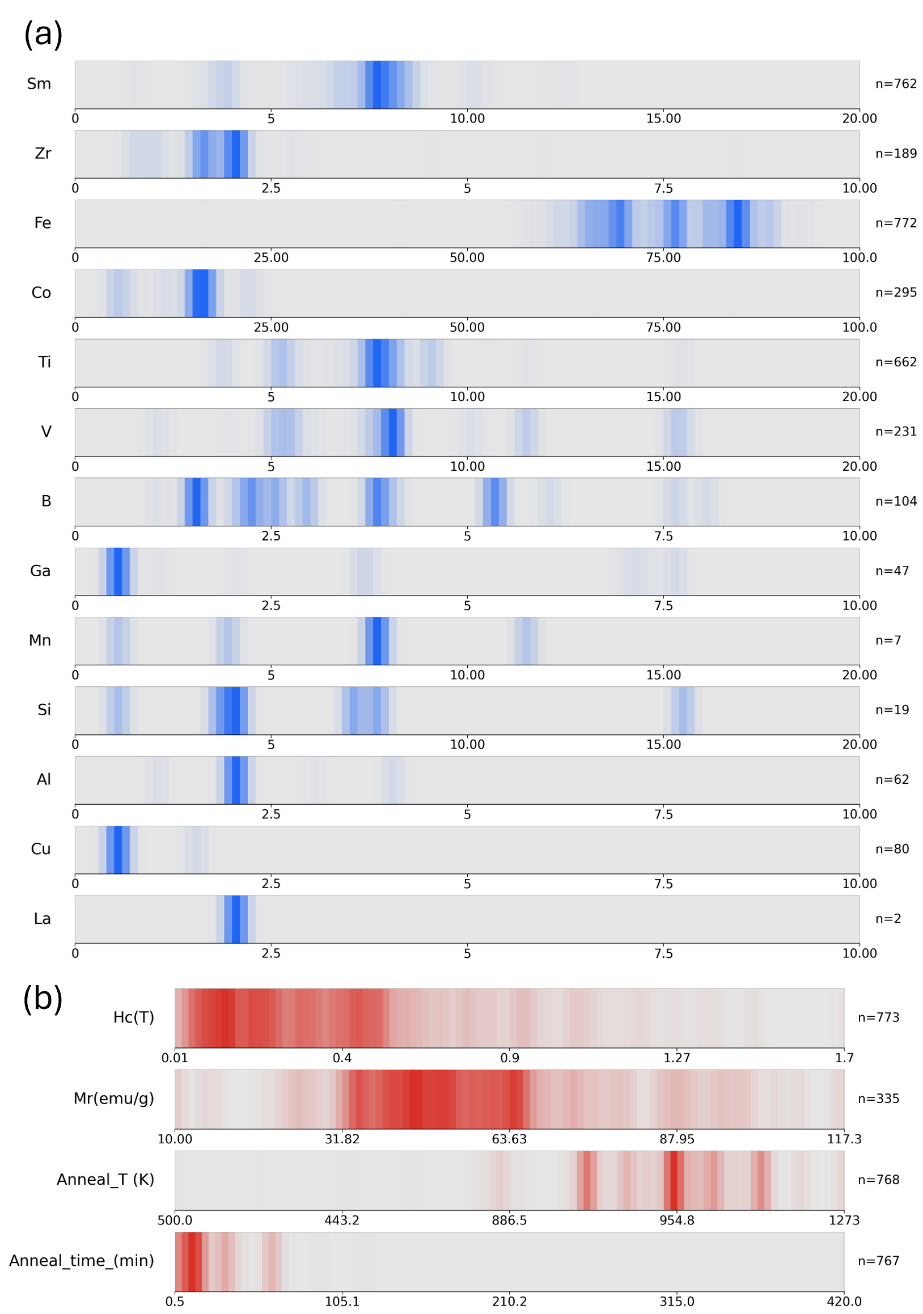
*

**Figure S1.** Overview of the distribution of features in the dataset including (a) elemental composition and (b) *μ*_0_*H*_c_, *M*_r_, annealing temperature, and annealing time (from top to bottom). For each parameter, a total number of non-zero values in the dataset is indicated on the right-hand side.


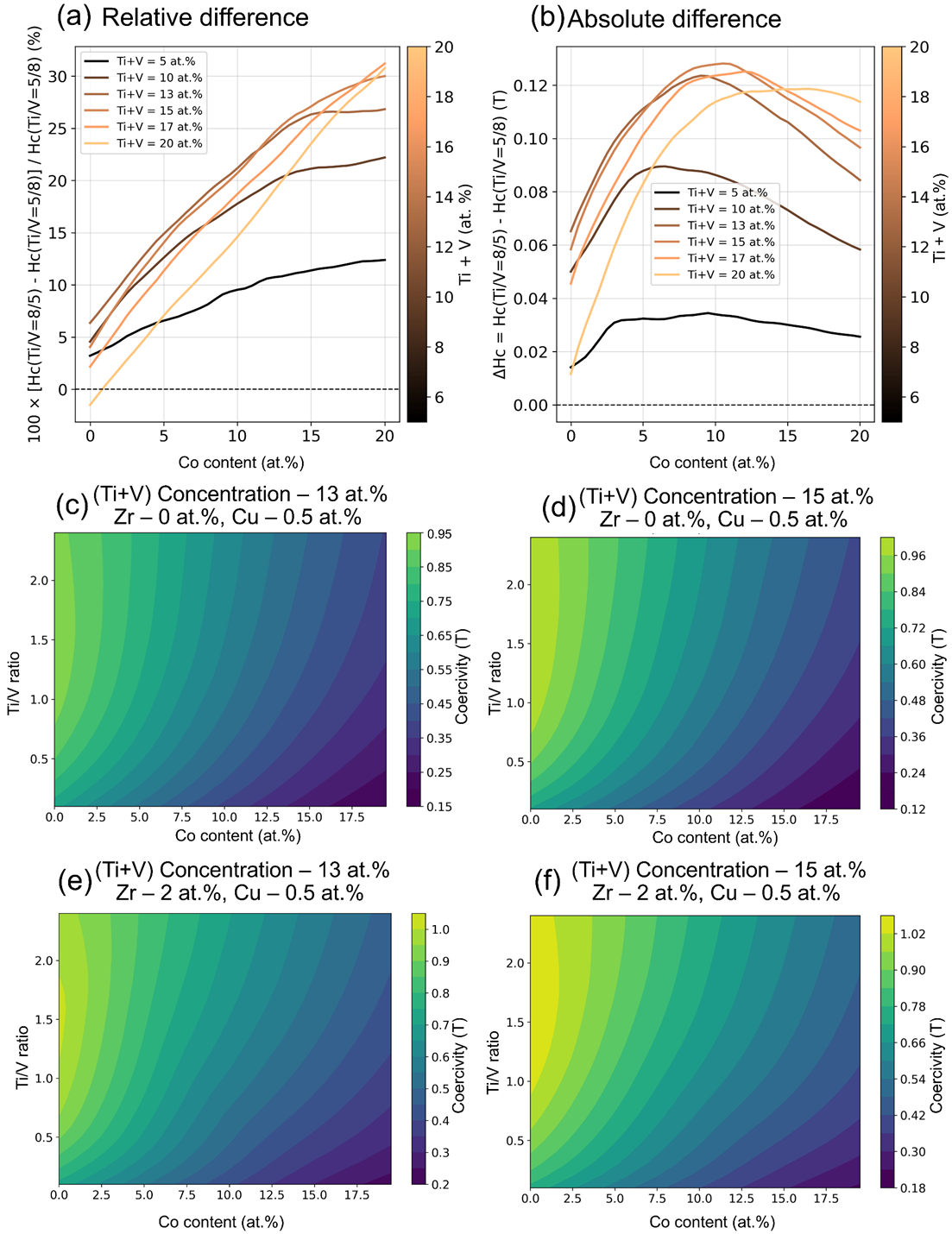


**Figure S2.** Predicted coercivity maps for SmFe₁₂-based alloys as a function of Co content and Ti/V ratio. (a) Relative coercivity difference and (b) absolute coercivity difference between Ti-rich and Ti-lean samples with Ti/V = 8/5 and Ti/V = 5/8 as a function of Co content for different total Ti+V contents. The corresponding predicted coercivity maps are shown for (c) Ti+V = 13 at.%, Zr = 0 at.%, Cu = 0.5 at.%; (d) Ti+V = 15 at.%, Zr = 0 at.%, Cu = 0.5 at.%; (e) Ti+V = 13 at.%, Zr = 2 at.%, Cu = 0.5 at.%; and (f) Ti+V = 15 at.%, Zr = 2 at.% , Cu = 0.5 at.%,

**
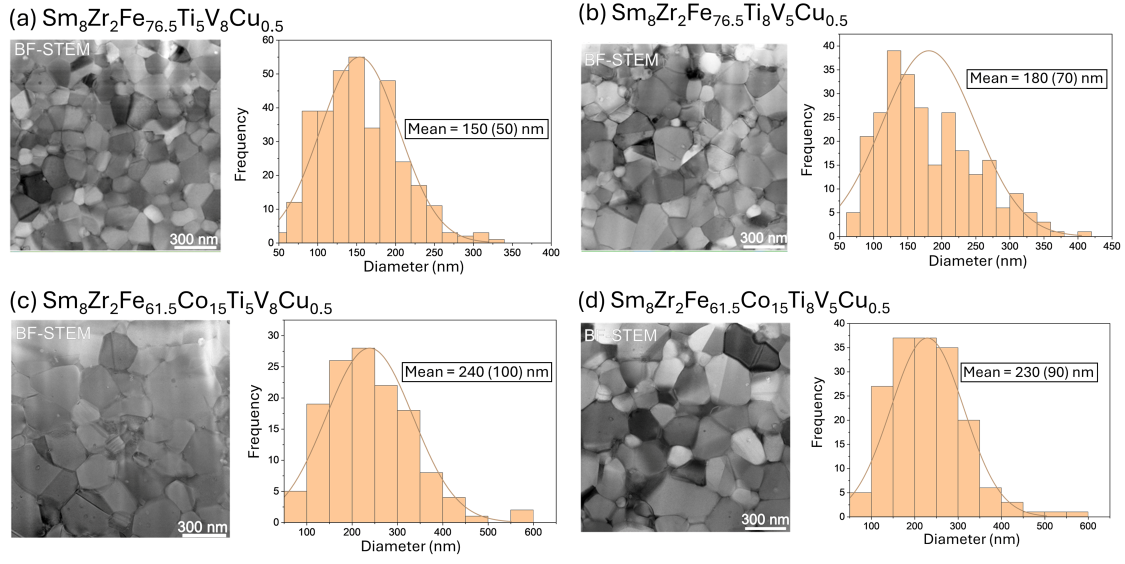
** **Figure S3.** BF–STEM micrographs and grain size distributions of melt-spun ribbons and their corresponding grain-size distributions for: (a) Sm_8_Zr_2_Fe_76.5_Ti_5_V_8_Cu_0.5_, (b) Sm_8_Zr_2_Fe_76.5_Ti_8_V_5_Cu_0.5_, (c) Sm_8_Zr_2_Fe_61.5_Co_15_Ti_5_V_8_Cu_0.5_, and (d) Sm_8_Zr_2_Fe_61.5_Co_15_Ti_8_V_5_ Cu_0.5_.


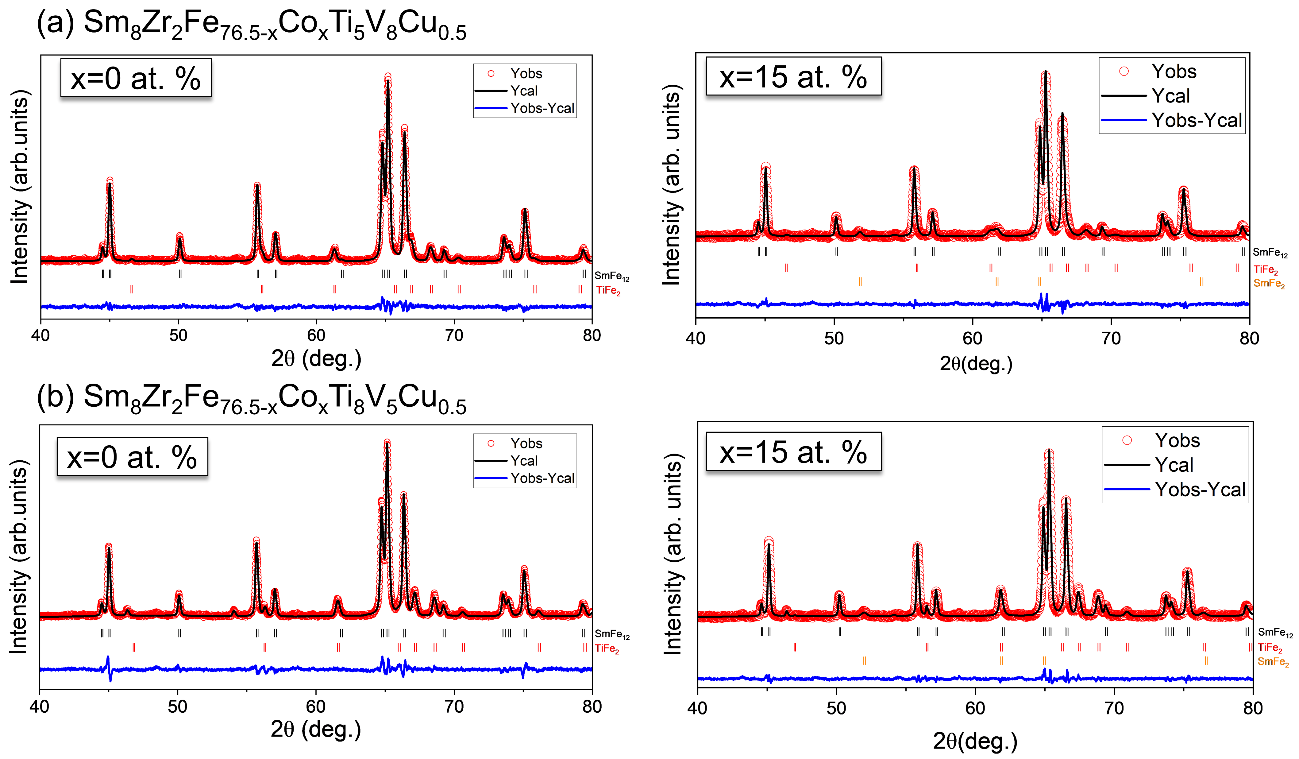


**Figure S4.** Rietveld refinement from XRD patterns of (a) Ti-lean (Sm_8_Zr_2_Fe_76.5-x_Co_x_Ti_5_V_8_Cu_0.5_) and (b) Ti-rich (Sm_8_Zr_2_Fe_76.5-x_Co_x_Ti_8_V_5_Cu_0.5_) ribbons with varying Co content (x = 0 and 15 at.%). Red circles are the observed data (Yobs), black lines the calculated pattern (Ycalc), and blue lines the difference (Yobs–Ycalc). Both compositions show the dominant presence of the SmFe_12_ phase, while minor phases such as SmFe_2_ and Fe_2_Ti are also detected.

**Table S1.** Phase weight fractions (vol.%) of SmFe_12_, Fe_2_Ti, and SmFe_2_ in the Ti-rich and Ti-lean ribbons for the Co-free and 15 at.% Co addition, quantified by Rietveld refinement of the XRD patterns (fig. S3).

| Samples | | Concentrations (vol.%) | | |
| --- | --- | --- | --- | --- |
|  |  | SmFe_12_ | Fe_2_Ti | SmFe_2_ |
| Ti-rich | Co-free | 84.75 | 15.25 | 0 |
|  | 15 at.% Co | 84.60 | 10.32 | 4.46 |
| Ti-lean | Co-free | 86.78 | 13.22 | 0 |
|  | 15 at.% Co | 87.45 | 9.44 | 3.11 |

**
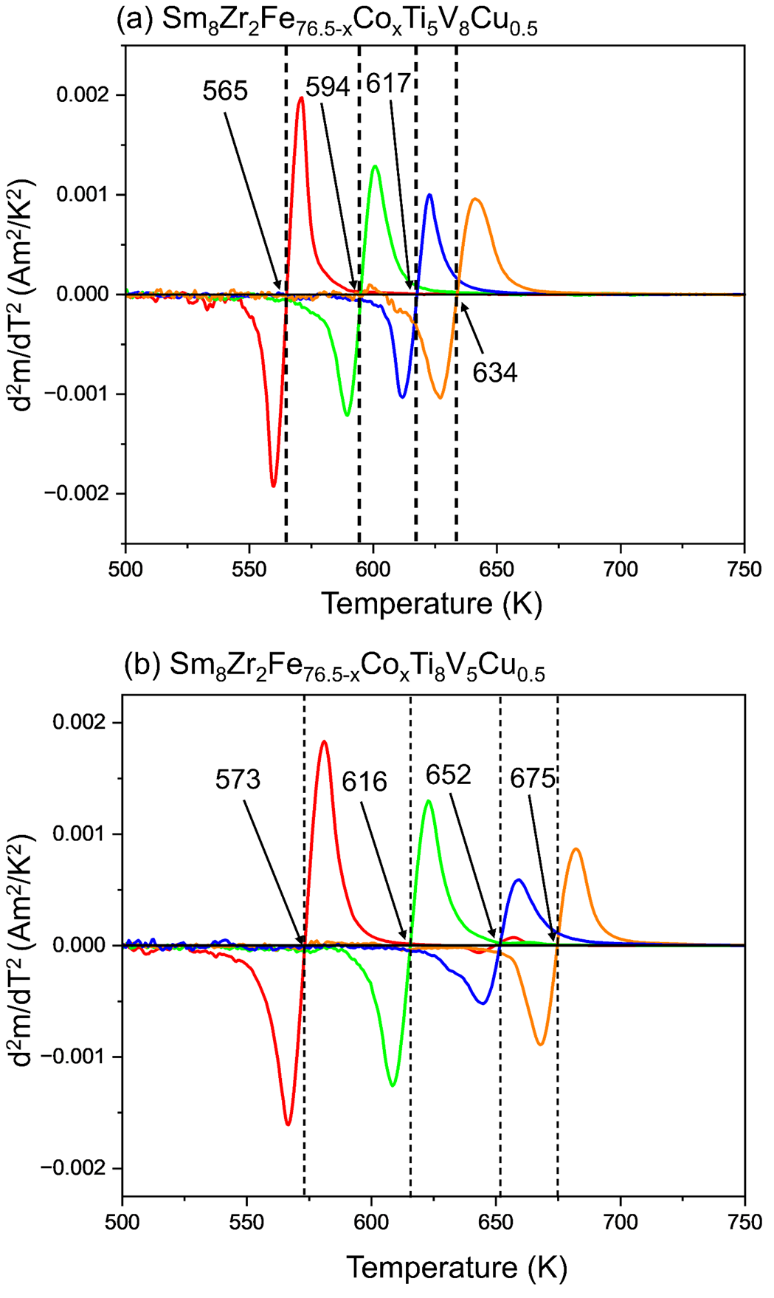
**

**Figure S5.** Second derivative d2M/dT2, for estimating the curie temperature (*T*_c_) of (a) Sm_8_Zr_2_Fe_76.5-x_Co_x_Ti_5_V_8_Cu_0.5_ and (b) Sm_8_Zr_2_Fe_76.5-x_Co_x_Ti_8_V_5_Cu_0.5_ ingots with x = 0, 5.5, 11, 15 at.% Co. The dashed vertical lines indicate the *T*_c_ values.

**
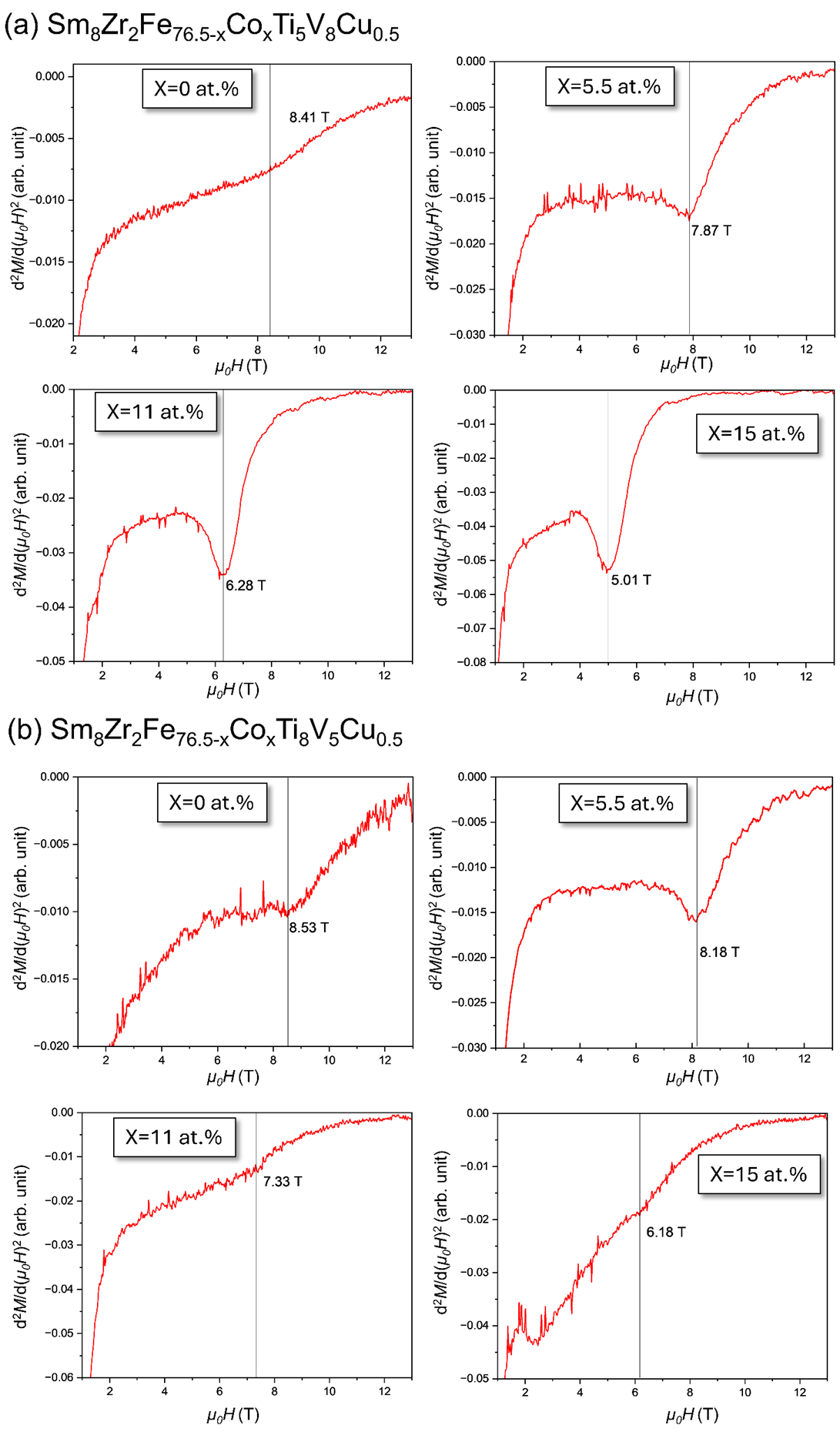
aa**

**Figure S6.** Second-derivative SPD profiles, (d^2^*M*/d(*μ*_0_*H*)^2^), obtained from the high-field magnetization curves of (a) Sm_8_Zr_2_Fe_76.5-x_Co_x_Ti_5_V_8_Cu_0.5_ and (b) Sm_8_Zr_2_Fe_76.5-x_Co_x_Ti_8_V_5_ Cu_0.5_ melt-spun ribbons. The vertical grey lines mark the singular points used to estimate the anisotropy field.


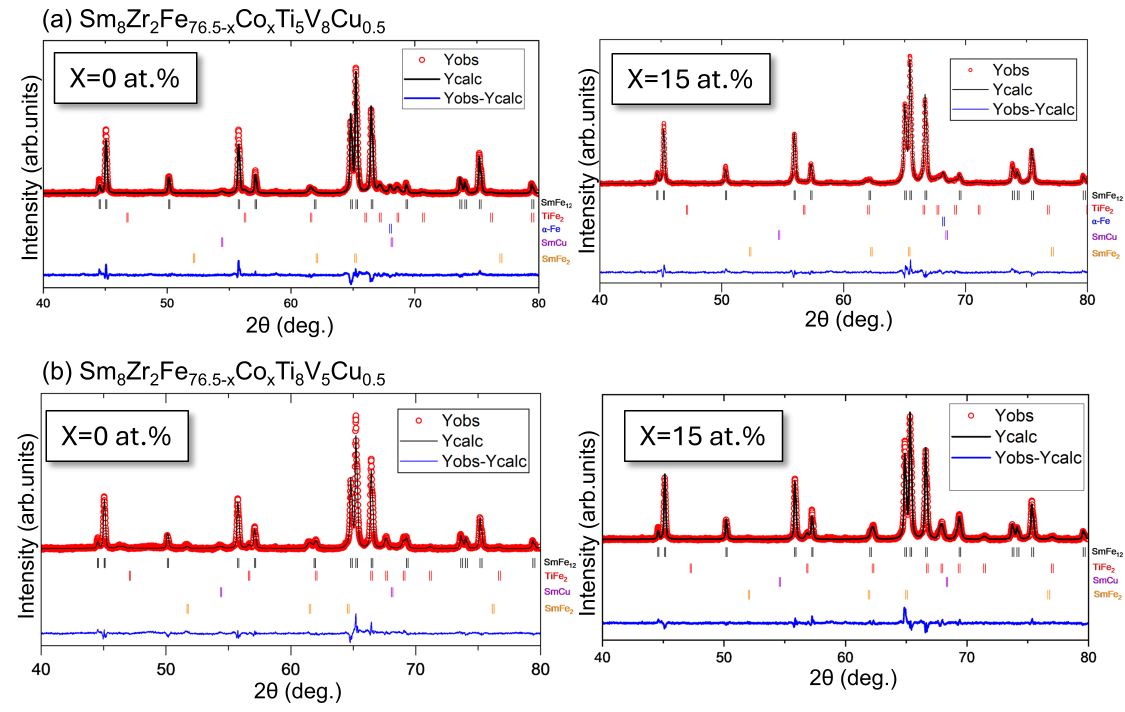


**Figure S7.** Rietveld refinement fits for ingot samples: (a) Sm_8_Zr_2_Fe_76.5-x_Co_x_Ti_5_V_8_Cu_0.5_ and (b) Sm_8_Zr_2_Fe_76.5-x_Co_x_Ti_8_V_5_Cu_0.5,_ where x = 0 and 15. Red circles are the observed data (Yobs), black lines the calculated pattern (Ycalc), and blue lines the difference (Yobs–Ycalc). Colored tick marks below each pattern represent the Bragg positions of SmFe₁₂, TiFe₂, α-Fe, SmCu, and SmFe₂. The close overlap of observed and calculated curves demonstrates excellent fit quality and confirms the identified phases.


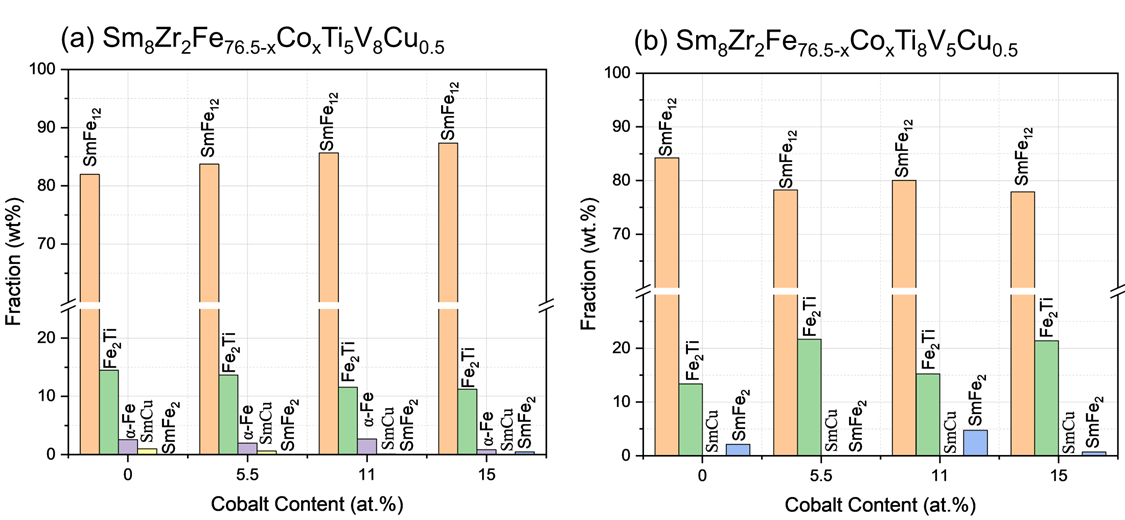


**Figure S8.** Phase fractions (wt%) from Rietveld refinement of ingot (a) Sm_8_Zr_2_Fe_76.5-x_Co_x_Ti_5_V_8_Cu_0.5_ and (b) Sm_8_Zr_2_Fe_76.5-x_Co_x_Ti_8_V_5_Cu_0.5_, x = 0, 5.5, 11, 15 at.% Co. The 1:12 phase dominates in all samples, Trace amounts of Fe_2_Ti, α‑Fe, SmCu, and SmFe_2_ are also present.
